# An observational, prospective, multicenter study on rescue high-frequency oscillatory ventilation in neonates failing with conventional ventilation

**DOI:** 10.1371/journal.pone.0217768

**Published:** 2019-06-10

**Authors:** Omer Erdeve, Emel Okulu, Gaffari Tunc, Yalcın Celik, Ugur Kayacan, Merih Cetinkaya, Gokhan Buyukkale, Hilal Ozkan, Nilgun Koksal, Mehmet Satar, Mustafa Akcali, Canan Aygun, Servet Ozkiraz, Umut Zubarioglu, Sezin Unal, Hatice Turgut, Kurthan Mert, Tulin Gokmen, Barıs Akcan, Begum Atasay, Saadet Arsan, Rescue-HFOV Trial Group

**Affiliations:** 1 Division of Neonatology, Department of Pediatrics, Ankara University School of Medicine, Ankara, Turkey; 2 Division of Neonatology, Department of Pediatrics, Mersin University School of Medicine, Mersin, Turkey; 3 Department of Neonatology, University of Health Sciences, Kanuni Training and Research Hospital, Istanbul, Turkey; 4 Division of Neonatology, Department of Pediatrics, Uludag University School of Medicine, Bursa, Turkey; 5 Division of Neonatology, Department of Pediatrics, Cukurova University School of Medicine, Adana, Turkey; 6 Division of Neonatology, Department of Pediatrics, Ondokuz Mayıs University School of Medicine, Samsun, Turkey; 7 Neonatal Intensive Care Unit, Medicalpark Hospital, Gaziantep, Turkey; 8 Department of Neonatology, University of Health Sciences, Sisli Etfal Training and Research Hospital, Istanbul, Turkey; 9 Department of Neonatology, University of Health Sciences, Etlik Zubeyde Hanim Maternity Training and Research Hospital, Ankara, Turkey; 10 Division of Neonatology, Department of Pediatrics, Inonu University School of Medicine, Malatya, Turkey; 11 Neonatal Intensive Care Unit, Adana Numune Training and Research Hospital, Adana, Turkey; 12 Department of Neonatology, University of Health Sciences, Zeynep Kamil Training and Research Hospital, Istanbul, Turkey; 13 Division of Neonatology, Department of Pediatrics, Adnan Menderes University School of Medicine, Aydin, Turkey; Hopital Robert Debre, FRANCE

## Abstract

**Background:**

To achieve gas exchange goals and mitigate lung injury, infants who fail with conventional ventilation (CV) are generally switched to high-frequency oscillatory ventilation (HFOV). Although preferred in many neonatal intensive care units (NICUs), research on this type of rescue HFOV has not been reported recently.

**Methods:**

An online registry database for a multicenter, prospective study was set to evaluate factors affecting the response of newborn infants to rescue HFOV treatment. The study population consisted of 372 infants with CV failure after at least 4 hours of treatment in 23 participating NICUs. Patients were grouped according to their final outcome as survived (Group S) or as died or received extracorporeal membrane oxygenation (ECMO) (Group D/E). Patients’ demographic characteristics and underlying diseases in addition to their ventilator settings, arterial blood gas (ABG) analysis results at 0, 1, 4, and 24 hours, type of device, ventilation duration, and complications were compared between groups.

**Results:**

HFOV as rescue treatment was successful in 58.1% of patients. Demographic and treatment parameters were not different between groups, except that infants in Group D/E had lower birthweight (BW) (1655 ± 1091 vs. 1858 ± 1027 g, *p* = 0.006), a higher initial FiO_2_ setting (83% vs. 72%, *p* < 0.001), and a higher rate of nitric oxide exposure (21.8% vs. 11.1%, *p* = 0.004) in comparison to infants who survived (Group S). The initial cut-offs for a successful response on ABG were defined as pH >7.065 (OR: 19.74, 95% CI 4.83–80.6, *p* < 0.001), HCO_3_ >16.35 mmol/L (OR: 1.06, 95% CI 1.01–1.1, *p* = 0.006), and lactate level <3.75 mmol/L (OR: 1.09%95 CI 1.01–1.16, *p* = 0.006). Rescue HFOV duration was associated with retinopathy of prematurity (*p* = 0.005) and moderate or severe chronic lung disease (*p* < 0.001), but not with patent ductus arteriosus or intraventricular hemorrhage, in survivors (*p* > 0.05).

**Conclusion:**

Rescue HFOV as defined for this population was successful in more than half of the patients with CV failure. Although the response was not associated with gestational age, underlying disease, device used, or initial MV settings, it seemed to be more effective in patients with higher BW and those not requiring nitric oxide. Initial pH, HCO_3_, and lactate levels on ABG may be used as predictors of a response to rescue HFOV.

## Introduction

The use of conventional ventilation (CV) in newborn infants with respiratory failure saves lives, but its use is associated with lung injury and chronic lung disease (CLD). A newer form of ventilation, high-frequency oscillatory ventilation (HFOV), has been shown to result in less lung injury in both experimental and clinical studies [1,2]. As HFOV has been suggested as a useful element of lung protection strategies to achieve gas exchange goals in addition to mitigation of lung injury, infants who fail with CV are generally switched to HFOV in many neonatal intensive care units (NICUs) [3–5]. Despite its widespread adoption, especially in NICUs without extracorporeal membrane oxygenation (ECMO) capability, there have been no recent reports regarding this type of rescue HFOV.

Recent Cochrane meta-analyses on rescue HFOV in newborn infants were published in 2000 and 2001, and the numbers of studies included were too small to allow for definitive conclusions [6,7]. Indeed, randomized controlled multicenter trials in older children and adults indicated that use of HFOV was not associated with decreased mortality [3,8,9]. These trials in older age groups resulted in uncertainty regarding the use of rescue HFOV in NICUs, where it is commonly used. Therefore, we aimed to establish a prospective online registry database to evaluate the factors that affect the response to rescue HFOV in newborn infants with CV failure during respiratory management. To facilitate identification of risk-reduction strategies, we defined the parameters to estimate the response of a neonate to rescue HFOV.

## Materials and methods

### Settings and patients

After the establishment of the Rescue HFOV Online Registry in May 2016, a multicenter prospective observational cohort study was conducted among infants who were born at gestational week (GW) ≥24 and switched to HFOV. Clinical directors in NICUs nationwide were made aware of the study, and 23 NICUs participated. The NICUs were asked to add all hospitalized patients who received rescue HFOV for at least 4 hours to the registry database daily using an online standard, patient-specific electronic case report form (eCRF). Data were collected prospectively and entered by trained neonatologists. The study was approved by the Online Studies Scientific Steering Committee of the Turkish Neonatal Society and by the Institutional Review Board of Ankara University.

Infants who were receiving CV but still had respiratory failure and were switched to rescue HFOV for at least 4 hours were enrolled in the study. Demographic data, including gestational age (GA), birthweight (BW), gender, delivery mode, diagnosis on admission, and pregnancy history, were recorded. Postnatal age at the time of intubation, surfactant history, time on CV, postnatal age at the time of switching to rescue HFOV, type of device used, and duration of rescue HFOV were obtained. Patients were also checked for inhaled nitric oxide (iNO) and ECMO records. All patients were monitored with continuous pulse oximetry and arterial blood gas (ABG) analysis measurements. Data were prospectively recorded prior to HFOV (0 hours) and at 1, 4, and 24 hours of rescue HFOV. An umbilical arterial catheter was generally used for rapid ABG analysis. The oxygenation index (OI) [OI = Mean alveolar pressure (MAP)·FiO_2_·100/PaO_2_] was calculated prior to and after HFOV treatment.

Patients were grouped according to their final responses to rescue HFOV treatment as Survived (Group S) and Died or received ECMO (Group D/E). Patients’ demographic characteristics and underlying diseases, in addition to their ventilator settings, ABG analysis results, type of device, and ventilation duration, were compared between groups. The associations of rescue HFOV duration with morbidities such as CLD, retinopathy of prematurity (ROP), patent ductus arteriosus (PDA), and grade III–IV intraventricular hemorrhage (IVH) were evaluated in survivors.

### Respiratory support

All patients were treated with CV using volume-controlled ventilation before beginning rescue HFOV. Tidal volume was kept below 7 ml/kg. Hypercarbia was tolerated if arterial pH was above 7.25. HFOV was initiated in cases in which the FiO_2_ requirement exceeded 0.6 to maintain an arterial oxygen saturation of >90%. A high-volume strategy consisting of incremental increases in MAP until arterial oxygen saturation reached >90% with FiO_2_ <0.6 and avoiding lung overdistension was employed. At a minimum, chest radiography was performed during CV, at 2–4 hours of rescue HFOV, and then every 12–24 hours, and interpreted for the presence or absence of lung hyperinflation and air leakage. Lung overdistension was identified by regular chest radiography and was defined as presence of more than nine posterior ribs of lung expansion. Oscillation of pressure amplitude was initially adjusted to provide adequate chest wall movement and was subsequently titrated to maintain PaCO_2_ between 35 and 50 mmHg. The frequency was initially set at 6–15 Hz with an inspiratory time of 33%. During rescue HFOV, patients were generally sedated but not immobilized with muscle paralysis. iNO was available in all centers participated in the study, and it was used for neonates ≥34 weeks’ gestational age who fail to respond to appropriate respiratory management with PaO_2_ <100 mmHg on FiO_2_ 1.0 and an OI >25 [10]. Newborns with severe hypoxic respiratory failure, refractory to maximal medical management, and a potentially reversible etiology were referred to receive ECMO according to the criteria reported by the Extracorporeal Life Support Organization (ELSO), but only in one center where ECMO is available [11].

The weaning process was initiated once FiO_2_ reached <0.4. MAP was gradually decreased by 1–2 cmH_2_O, and the oscillation pressure amplitude was adjusted to maintain PaCO_2_ between 35 and 50 mmHg. Extubation was considered when the patient’s condition had been stable for at least 12 hours if adequate oxygenation could be maintained with FiO_2_ <0.3 and MAP <8 cmH_2_O.

### Statistical analysis

The data were analyzed with SPSS for Windows 15 (SPSS, Chicago, IL). Descriptive statistics are summarized as the mean (SD) for continuous variables and number (percentage) for categorical variables. The significance of differences was examined by *t*-test and the Mann–Whitney U-test. Nominal variables were assessed using Pearson’s chi-squared test or Fisher’s exact test. Comparisons of gas exchange variables over time were performed by the Friedman rank-sum procedure, a paired nonparametric statistic, and two-tailed Wilcoxon’s matched-pairs test. Spearman’s correlation coefficient was used to study the trends of these parameters during the rescue HFOV trial. Parameters were subjected to receiver operating characteristic (ROC) curve analysis if they carried a distinctive feature for mortality. Threshold values according to the Youden index were calculated for the variables with distinctive features. The value at the highest sensitivity and selectivity was determined as the threshold value. In all analyses, *p* < 0.05 was taken to indicate statistical significance.

## Results

### Demographic data of the study group

**Fig 1:**
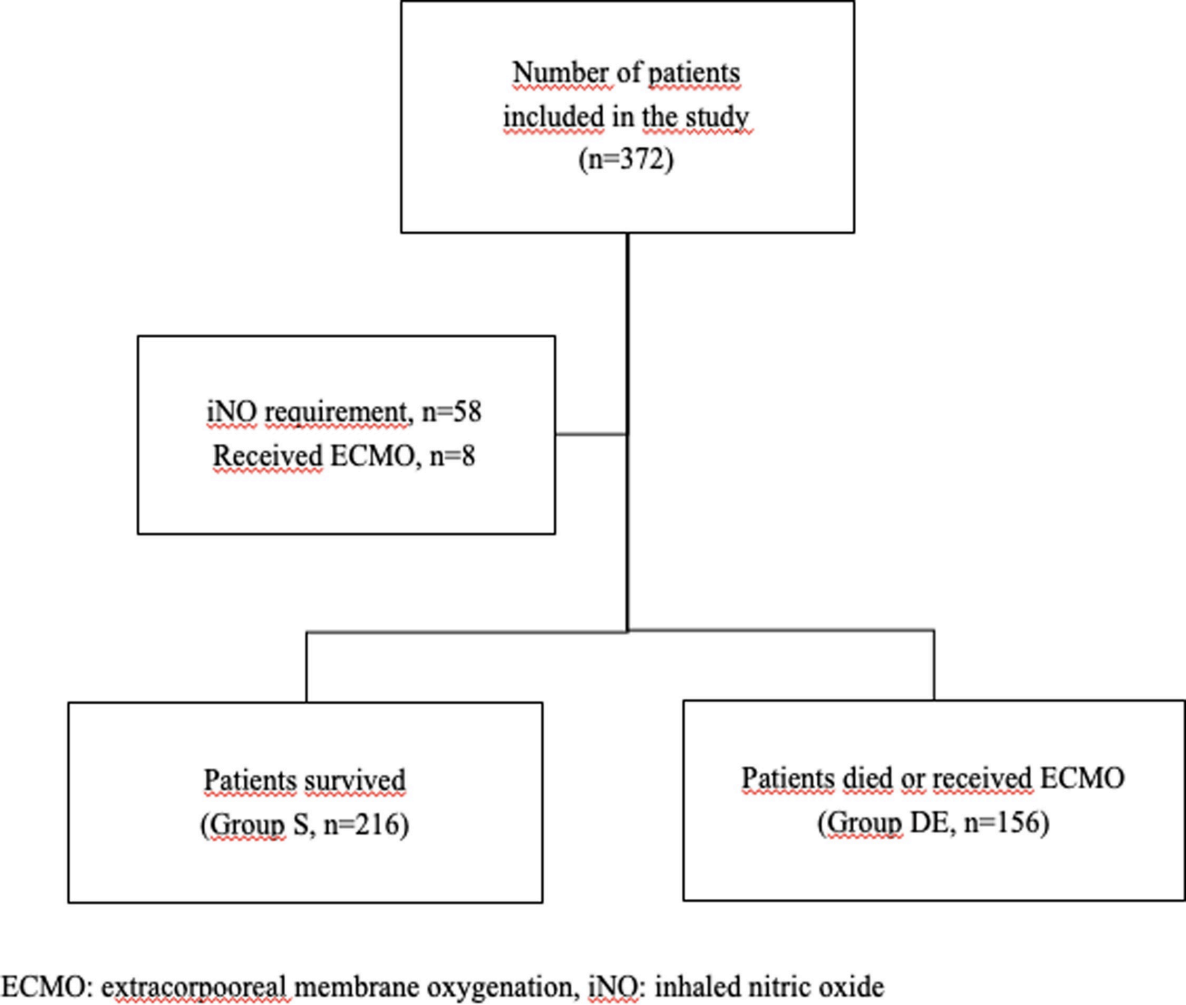
Flow chart of the study.

Between May 2016 and October 2017, 372 newborn infants with a mean GA of 36.1 ± 5.1 weeks and mean BW of 1773 ± 1058 g were enrolled (Fig 1). The male to female ratio was 1.7. Most of the patients (79%) were delivered by cesarean section. The major diseases requiring rescue HFOV were listed as respiratory distress syndrome (47%), congenital pneumonia (12%), sepsis (9%), congenital diaphragmatic hernia (8%), meconium aspiration syndrome (6%), and persistent pulmonary hypertension (5%). The rest of the patients (13%) were air leak syndrome (n = 14), perinatal asphyxia (n = 10), bronchopulmonary dysplasia (n = 9), pulmonary hemorrahage (n = 8) and pleural effusion (n = 6).

### Data related to rescue HFOV support

Among the total study population of 372 patients, 58% (*n* = 216) survived (Group S), and 42% (*n* = 156) died or received ECMO (Group D/E). Eight (2.2%) patients received ECMO support, among whom only three survived. The mean GA, postnatal intubation time (h), duration of CV prior to HFOV (h), duration of ventilation on HFOV (h), rate of surfactant use, and gender were not significantly different between groups (*p* > 0.05). Group D/E had lower mean BW (*p* = 0.067) in addition to a shorter hospitalization period (*p* = 0.001) and higher rate of iNO exposure (*p* = 0.004). As initial settings of HFOV, the median ΔP and MAP (cmH_2_O) were not different between groups, whereas the mean frequency (Hz) was higher in Group S (*p* = 0.011), and the median FiO_2_ was higher in Group D/E (*p* < 0.001) (Table 1).

**Table 1:** The comparison of groups according to clinical findings.

	Group S	Group DE	p
	(n = 216)	(n = 156)	
Gestational age (w)*	32±4.9	31.1±5.4	0.067
Birth weight (g)*	1,858±1,027	1,655±1,091	0.006
**Gender (male), n (%)**	146 (67.6)	90 (57.7)	0.06
**Type of delivery (CS^a^), n (%)**	174 (80.6)	119 (76.3)	0.32
**Surfactant use, n (%)**	167 (77.3)	124 (79.5)	0.7
**iNO^b^ use, n (%)**	24 (11.1)	34 (21.8)	0.004
**Age at postnatal intubation (h)^†^**	4 (0–576)	1 (1–312)	0.87
**Age at switched to HFOV^c^ (h)^†^**	37.5 (1–1,081)	41 (1–4,504)	0.48
**Duration of CV^d^ prior to HFOV (h)^†^**	30 (1–1,080)	27 (1–4,503)	0.81
**HFOV settings**			
Frequency (Hz)*	10.2±1.8 (6–15)	9.7±2.2	0.011
***Delta P*^†^**	27 (12–50)	28.5 (11–50)	0.19
***MAP^e^ (cmH_2_O)^†^***	12 (8–22)	13 (7–22)	0.55
***FiO_2_*^†^**	70 (21–100)	100 (21–100)	< 0.001
Duration on HFOV (h)*	116±178.7	132.1±144	0.2
Length of hospitalization (d)*	53.7±34.3	20±24.7	0.001

^a^CS: cesarean section;

^b^iNO: inhaled nitric oxide;

^c^HFOV: high frequency oscillatory ventilation;

^d^CV: conventional ventilation;

^e^MAP: mean alveolar pressure

*Data given as mean ± SD,

^†^Data given as median (range)

### Changes in results of blood gas analysis

All patients in both groups (Group S, n = 216 and group DE, n = 156) had ABG analysis just prior to HFOV, at 1st and 4th hours of HFOV treatment. All patients in group S (n = 216) had ABG analysis at 24th hours of HFOV treatment, whereas only 5 patients from group DE did not have 24th hour ABG analysis.

**Fig 2:**
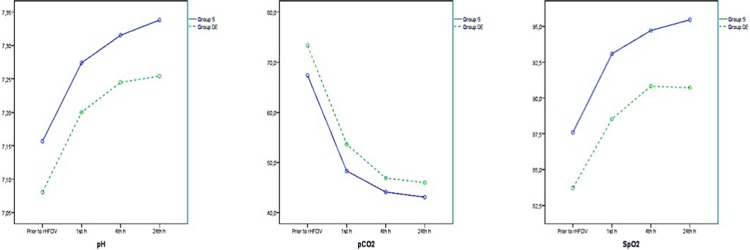
2A, 2B and 2C. Changes in pH, pCO_2_ and SpO_2_.

**Fig 3:**
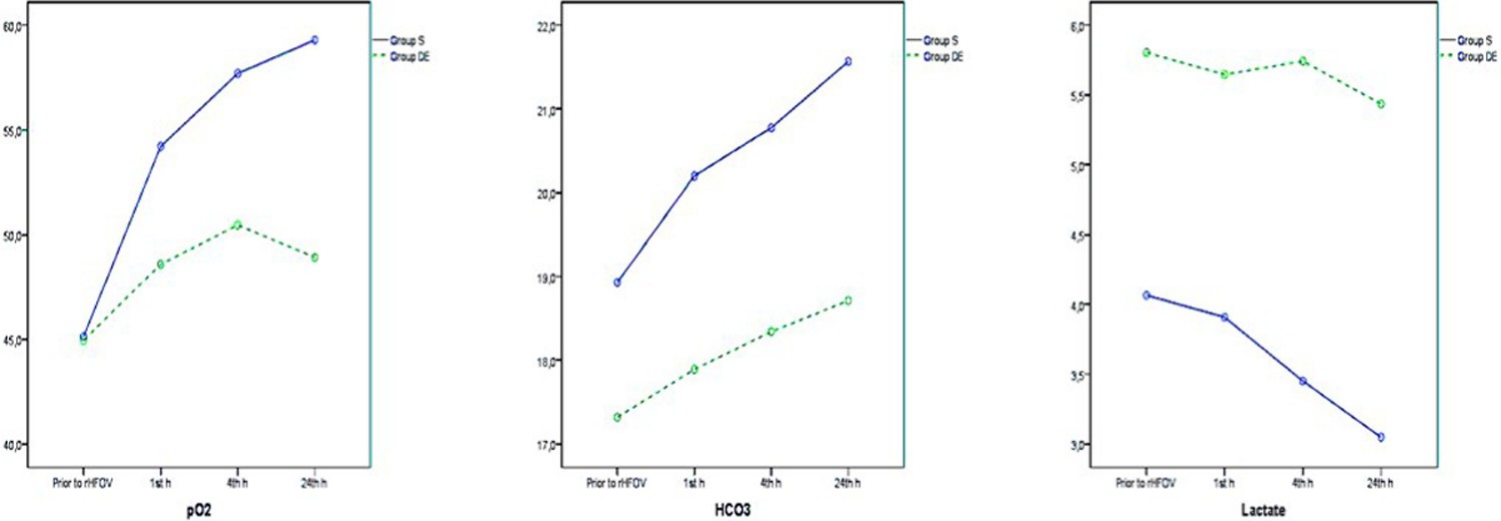
3A, 3B and 3C. Changes in pO_2_, HCO_3_ and lactate.

**Fig 4:**
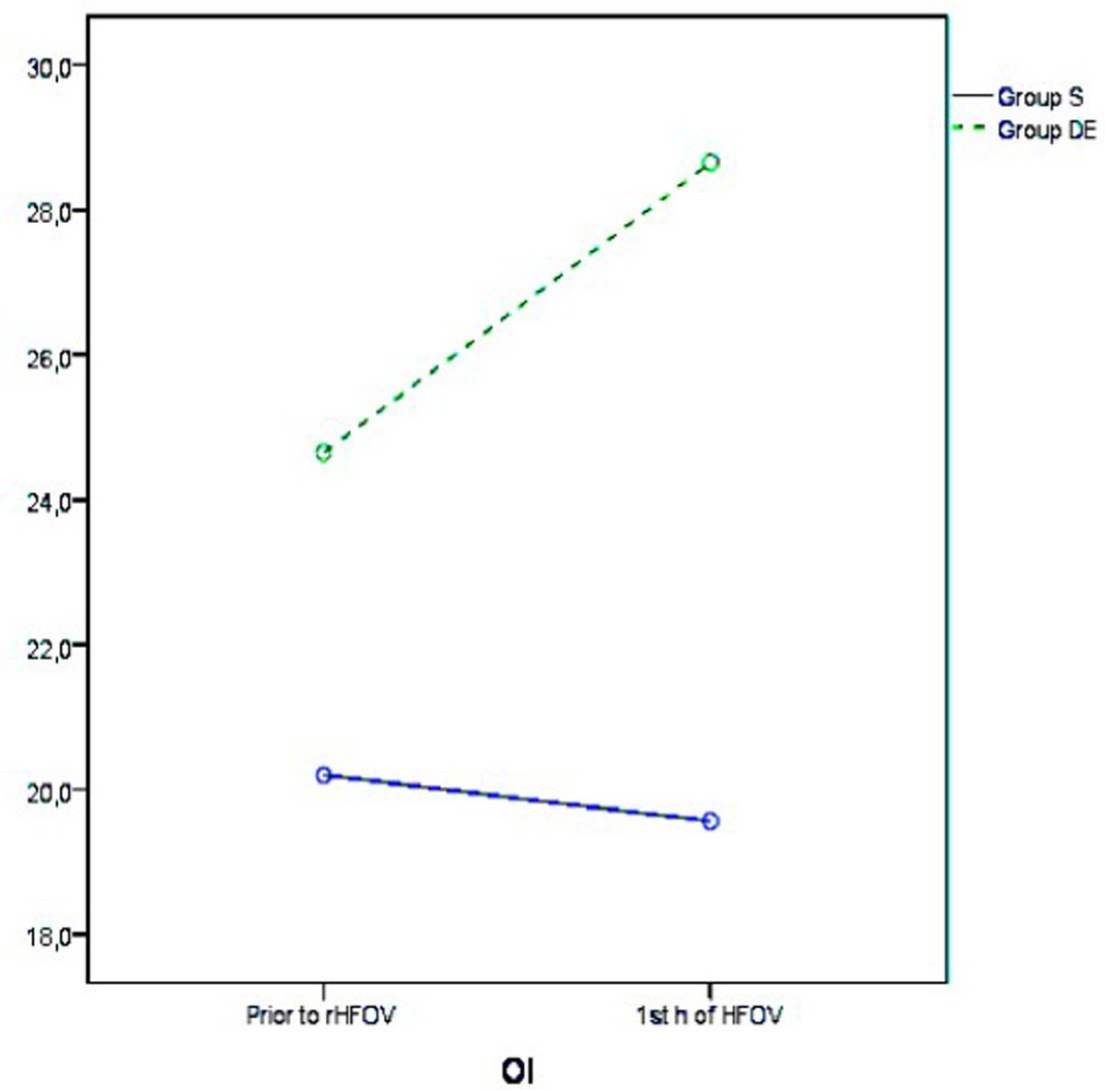
Evolution of OI.

HFOV treatment resulted in improvement in pH, pCO_2_, and SpO_2_ levels in both groups, and the significance of differences between groups was consistent across all ABG analysis results (Fig 2A, 2B and 2C and Table 2). Although HFOV treatment was associated with improvement in pO_2_, HCO_3_, and lactate levels in Group S, these improvements were not observed in Group D/E (Fig 3A, 3B and 3C and Table 3). The mean OI of group S improved with HFOV treatment, whereas the mean OI of group did not improve with HFOV (Fig 4 and Table 4).

**Table 2:** pH, pCO_2_ and SpO_2_ analysis of groups.

	pH				pCO_2_				SpO_2_			
	Group S (n = 216)		Group DE (n = 151)		Group S (n = 216)		Group DE (n = 151)		Group S (n = 216)		Group DE (n = 151)	
	*Mean ± SD*	*95% Cl*	*Mean ± SD*	*95% Cl*	*Mean ± SD*	*95% Cl*	*Mean ± SD*	*95% Cl*	*Mean ± SD*	*95% Cl*	*Mean ± SD*	*95% Cl*
**Prior to rHFOV**	7.16±0.14	7.14–7.18	7.08±0.17	7.06–7.1	67.4±23.8	64–70,7	73.3±26.3	69.3–77.3	87.6±6.4	86.5–88.7	83.7±9.9	82.4–85.0
**1st h**	7.27±0.11	7.25–7.29	7.2±0.17	7.18–7.22	48.3±13.3	46.1–50.5	53.6±19.9	51–56,2	93.1±4.9	92.1–94.0	88.5±9.3	87.4–89.7
**4 st h**	7.31±0.09	7.29–7.33	7.24±0.16	7.22–7.26	44.1±11.5	42.2–46	46.9±17.3	44.6–49.1	94.7±3.4	93.9–95.5	90.8±7.9	89.9–91.7
**24th h**	7.34±0.08	7.32–7.35	7.25±0.15	7.24–7.27	43.1±9.0	41.6–44.6	45.9±13.8	44.2–47.8	95.4±3.3	94.6–96.3	90.7±9.2	89.7–91.7

**Table 3:** pO_2_, HCO_3_ and lactate analysis of groups.

	pO_2_				HCO_3_				Lactate			
	Group S (n = 216)		Group DE (n = 151)		Group S (n = 216)		Group DE (n = 151)		Group S (n = 133)		Group DE (n = 79)	
	*Mean ± SD*	*95% Cl*	*Mean ± SD*	*95% Cl*	*Mean ± SD*	*95% Cl*	*Mean ± SD*	*95% Cl*	*Mean ± SD*	*95% Cl*	*Mean ± SD*	*95% Cl*
**Prior to rHFOV**	45.1±16.5	42.7–47.5	44.9±20.1	42.0–47.8	18.9±4.6	18.3–19.6	17.3±5.4	16.5–18.1	4.1±3.3	3.4–4.7	5.8±4.7	4.9–6.6
**1st h**	54.2±28.0	50.4–57.9	48.6±28.2	44.1–53.1	20.2±4.5	19.5–20.8	17.9±5.6	17.1–18.7	3.9±3.7	3.2–4.6	5.6±4.3	4.8–6.5
**4 st h**	57.7±30.2	53.6–61.7	50.5±30.3	45.6–55.3	20.8±3.7	20.2–21.4	18.3±5.4	17.6–19.0	3.4±3.1	2.8–4.0	5.7±4.2	4.9–6.5
**24th h**	59.3±28.1	55.7–62.3	48.9±23.9	44.7–53.1	21.6±3.6	21.0–22.1	18.7±5.2	18.0–19.4	3.1±3.0	2.4–3.6	5.4±4.1	4.7–6.2

**Table 4:** Evolution of OI with HFOV treatment.

	OI			
	Group S (n = 216)		Group DE (n = 151)	
	*Mean ± SD*	*95% Cl*	*Mean ± SD*	*95% Cl*
**Prior to rHFOV**	20.2±12.3	18.2–22.2	24.6±18.0	22.03.2027
**1st h of HFOV**	19.6±12.3	17.5–21.6	28.6±18.5	26.2–31.1

The cutoffs for successful response predictors on initial blood gas analysis prior to HFOV were defined as pH >7.065 (OR: 19.74, 95% CI 4.83–80.6, *p* < 0.001), HCO_3_ >16.35 mmol/L (OR: 1.06, 95% CI 1.01–1.1, *p* = 0.006), and lactate level <3.75 mmol/L (OR: 1.09, 95% CI 1.01–1.16, *p* = 0.006).

### Factors affecting mortality

The mortality rate at first-30 days was 79% (n = 124) in group DE (n = 156). Sixty-one percent (n = 96) of patients in group DE was born before 32 weeks’ gestation, 84 (87%) of 96 patients died before 36 weeks of corrected gestational age.

#### Gestational age

**Fig 5:**
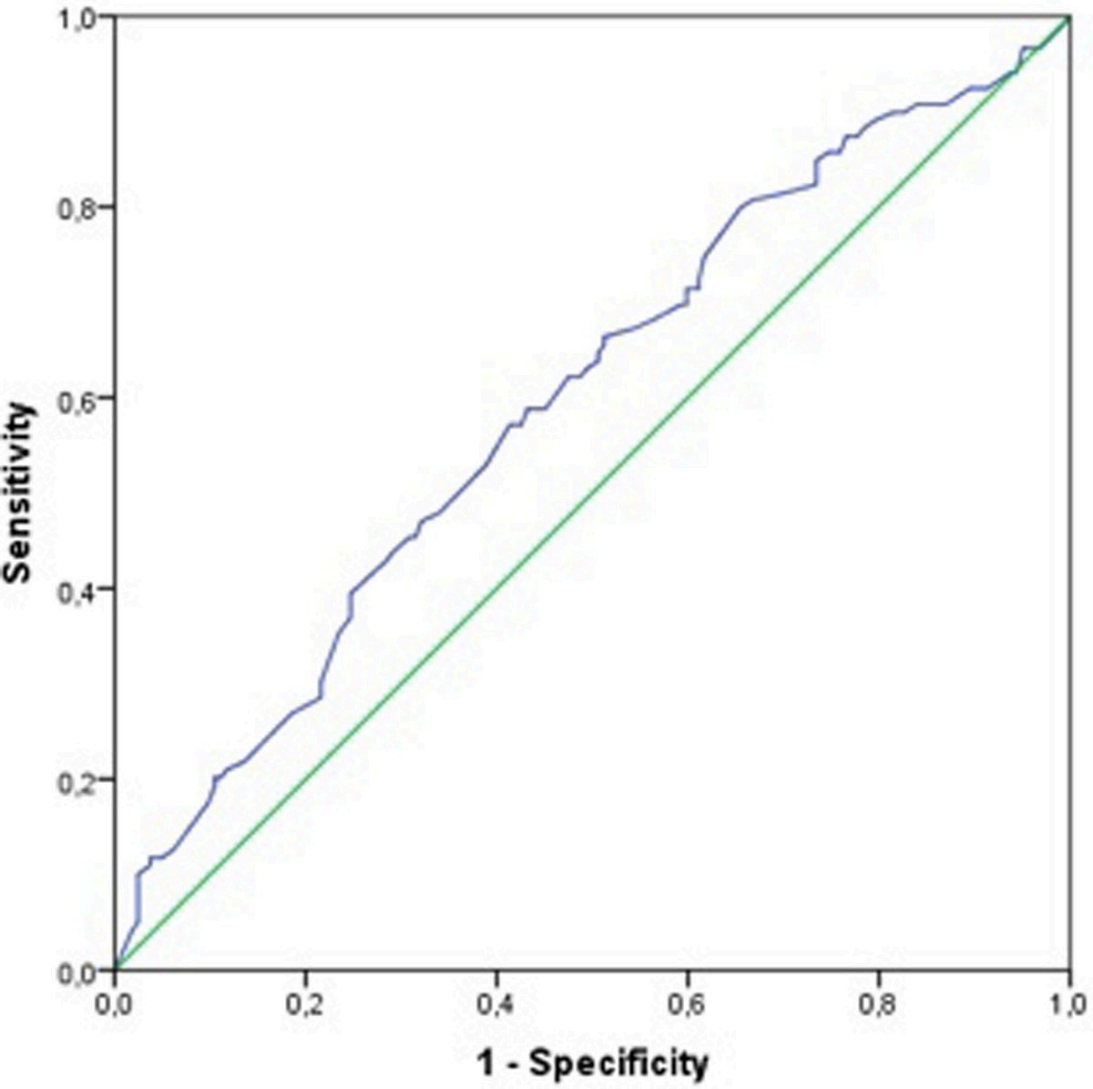
ROC curve for GW.

There were 281 patients born <37 weeks’ gestation, and 112 patients born <28 weeks’ gestation in the study population. Although GA was not different between groups, subgroup analysis of patients born at gestational week (GW) <37 indicated that the mean GA was lower for infants who died than for infants who survived (28.6 ± 3.6 vs. 29.8 ± 3.6 wk, respectively, *p* = 0.006). In terms of mortality and/or receipt of ECMO, the threshold for GA was GW 28, with 61% sensitivity and 53% specificity in newborn infants born <37 weeks’ gestation. The risk of mortality was 1.79 times higher in infants born at GW <28.0 (95% Cl 1.101–2.905, *p* = 0.018). Fig 5 shows the ROC curve for GW.

#### Birth weight

**Fig 6:**
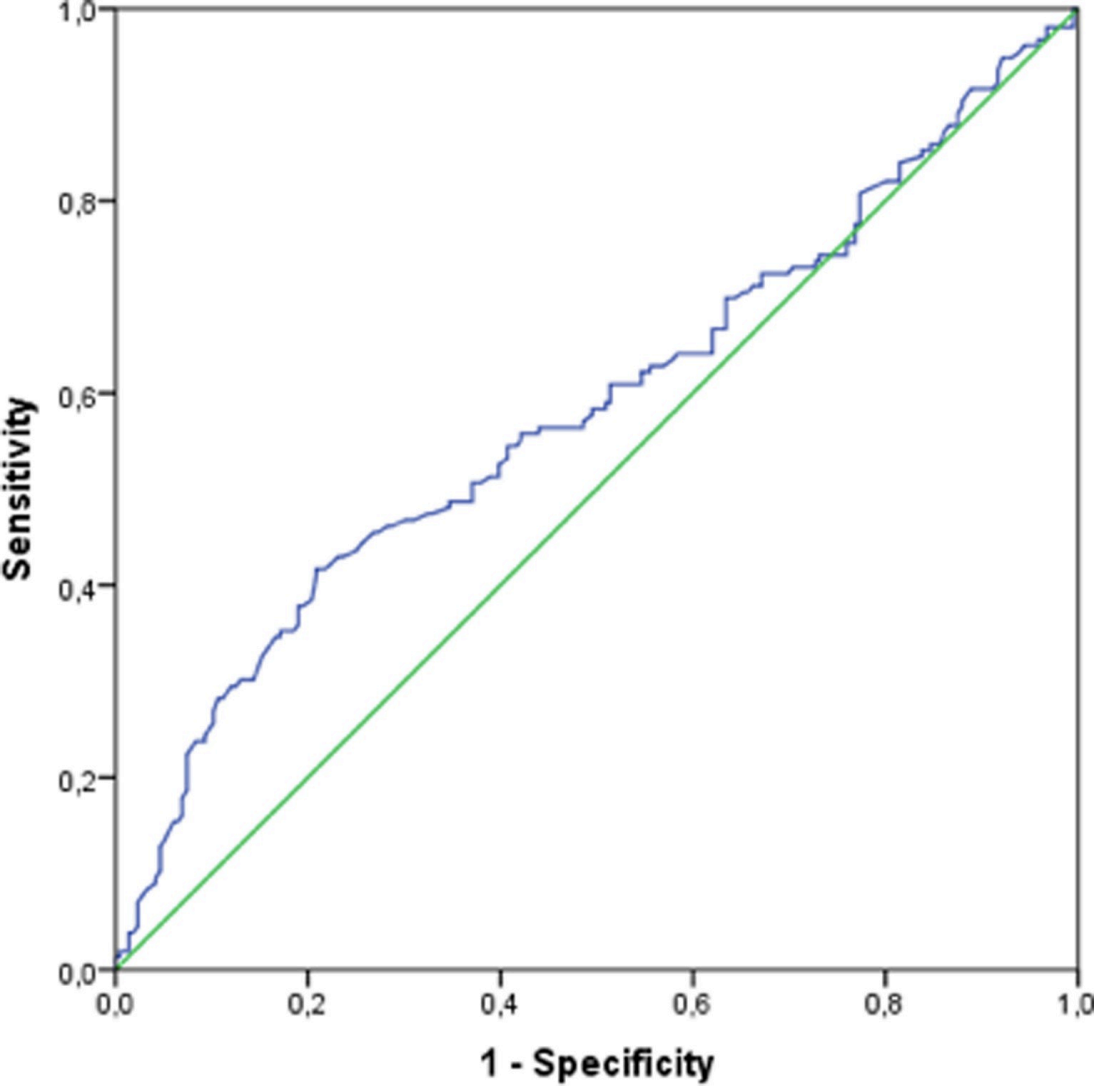
ROC curve for BW.

The mean BW was lower in group D/E than in group S (*p* = 0.006) (Table 1). With regard to mortality, the threshold for BW was 1405 g, with 56% sensitivity and 56% specificity. The risk of mortality was 1.6 times higher for infants with BW <1405 g (95% Cl 1.068–2.45, *p* = 0.023). Fig 6 shows the ROC curve BW.

#### Underlying disease

Table 5 shows the distrubition of underlying diseases according to the groups. HFOV treatment was more efficient in patient with congenital pneumonia, meconium aspiration syndrome, whereas more than half of the patients with congential diaphragmatic hernia did not benefit from rHFOV treatment (*p* = 0.038).

**Table 5:** The underlying diseases according to groups.

	Group S	Group DE
	(n = 216)	(n = 156)
**Respiratory distress syndrome, n (%)**	99 (56)	77 (44)
**Congenital pneumonia, n (%)**	34 (79)	9 (21)
**Sepsis, n (%)**	17 (50)	17 (50)
**Congenital diaphragmatic hernia, n (%)**	12 (43)	6 (57)
**Meconium aspiration syndrome, n (%)**	16 (70)	7 (30)
**Persistent pulmonary hypertension, n (%)**	9 (50)	9 (50)
**Other, n (%)**	29 (58)	21 (42)

#### Devices used for HFOV

The most common devices used for HFOV were the SLE 5000 (SLE, South Croydon, UK), Stephan Sophie (Stephan, Medizintechnik, Gakenbach, Germany), and Dräger Babylog 8000 plus (Drägerwerk AG & Co., Lübeck, Germany), which were used in 36%, 34.4%, and 6.7% of cases, respectively. There were no significant associations between mortality or receiving ECMO and the devices used for HFOV (*p* = 0.095)

### Factors affecting CLD, ROP, PDA, and IVH among survivors

Among survivors (n = 216), the incidences of CLD (moderate/severe), ROP, hemodynamically significant PDA, and Grade III–IV IVH were 18% (n = 39), 19% (n = 41), 30.6% (n = 66), and 4.6% (n = 10), respectively. CLD and ROP were correlated with the duration of rescue HFOV treatment (*p* < 0.001 and *p* = 0.005, respectively), whereas there were no correlations between the duration of rescue HFOV and PDA or IVH (*p* > 0.05). One-hundred-fifteen (53%) patient survived without any morbidity as CLD, ROP, PDA or IVH.

## Discussion

Given the paucity of data regarding the use of rescue HFOV in newborn infants with severe respiratory insufficiency and the issues of designing and conducting large randomized controlled trials in newborns, we performed a prospective multicenter observational cohort trial using data from a national database with a large sample size. Our results indicated that rescue HFOV was successful in more than half of all cases of CV failure in newborns as defined by response to oxygenation. Although the response was not associated with GA, underlying disease, the device used, or initial ventilator settings, HOFV seemed to be more effective in patients with higher BW and those not requiring nitric oxide. Initial pH, HCO_3_, and lactate levels on ABG may be used as predictors of a positive response. We believe that data obtained from our observational prospective study may be helpful in selecting patients for rescue HFOV or refer them for ECMO if it is available. Rescue HFOV seems to be safe except a weak association with ROP and CLD disease. Although we observed an association between ROP and HFOV, it is hard to talk about a correlation, because the lung disease was severe in all patients being submitted to rescue HFOV. FiO_2_ were high in this patients putting them at risk for ROP, but this might be the same when HFOV would not have been used as a rescue mode, i.e. it would be probably the same with CV only.

Combined experience with pediatric and adult HFOV use in respiratory insufficiency was promising for treatment until recently, and this encouraged NICUs to use this method in neonates [5,12,13]. This viewpoint changed with the results of two recent large randomized studies which concluded that the outcome was poorer for HFOV compared with CV in children with acute respiratory failure [8,9].

The first Cochrane meta-analysis on rescue HFOV in relation to newborns, which examined pulmonary dysfunction in preterm infants and was conducted in 2000, concluded that only one trial fulfilling the inclusion criteria showed a reduction in any new pulmonary air leakage. Pulmonary air leakage was prevented in only one in six infants by rescue HFOV. In contrast, there was one case of IVH of any grade for every six infants given rescue HFOV. Thus, there was a stronger but non-significant trend toward an increase in the incidence of grade 3 or 4 IVH. Therefore, this meta-analysis, which depended on a small amount of data, suggested that the harm may outweigh any benefit of rescue HFOV [7]. The next Cochrane meta-analysis on rescue HFOV versus CV for infants with severe pulmonary dysfunction born at or near term was published in 2001; again, only one trial met the inclusion criteria. This rescue trial with 81 infants showed no evidence of a reduction in mortality at 28 days or reduction in failed therapy on the assigned modality requiring crossover to the other modality. There were no differences in the number of patients requiring ECMO, days on a ventilator, days on oxygen therapy, or duration of hospitalization [6]. In addition, the ‘International, randomized, clinical HFOV versus conventional ventilation in infants with congenital diaphragmatic hernia’ (VICI) trial concluded that there was no statistically significant difference in combined outcome of mortality or CLD between CV and HFOV ventilation groups among infants with prenatally diagnosed congenital diaphragmatic hernia (CDH). In addition, a shorter ventilation time and reduced need for ECMO favored CV [14]. Finally, the most recent Cochrane meta-analysis in 2016 concluded that there was evidence that the use of elective HFOV compared with CV resulted in a small reduction in the risk of CLD, but the evidence was weakened by the inconsistency of this effect across trials [2]. However, in contrast to the results from these meta-analyses, which did not support the routine use of rescue HFOV, only small series have led to its widespread use for rescue in newborn infants with severe pulmonary dysfunction, especially in NICUs with no ECMO capability [15]. The main reason for this preference was improved gas exchange with rescue HFOV, presumably because of the more uniform saccular aeration, which also prevents lung injury [1].

ECMO, which requires complex and expensive equipment, is a standard treatment choice for respiratory failure in developed countries when all other treatment options have been exhausted, but it is available in only a few centers in Turkey [11]. Furthermore, transport on HFOV is difficult, and newborns in whom such ventilation has failed often do not tolerate being placed back on CV. The ability to distinguish between newborns who are likely to respond to HFOV and those who are likely to require ECMO, without delaying the safe use of HFOV, are challenges in the NICU [16]. In a series with 122 infants meeting the criteria for ECMO, De Lamos et al. demonstrated that the use of HFOV with an appropriate strategy decreased the need for ECMO in 53% of cases [17]. Our national series also suggested that rescue HFOV can be a step prior to ECMO.

In a retrospective study, Paranka et al. defined the presence of CDH/lung hypoplasia and lack of improvement in oxygenation after 6 hours of HFOV as major risk factors associated with failure of rescue HFOV [16]. Similarly, Jaballah et al. suggested early rescue intervention as an effective protocol for newborn infants >GW 34 weeks and concluded that treatment failure was associated with lack of improvement in oxygenation at 1 hour of HFOV [15]. Although we could not differentiate any specific disease for the response, guidelines now suggest CV instead of HFOV as first-line treatment in CDH [18]. The results of blood gas analyses in our study were compatible with previous studies indicating that serial oxygenation measurement may be used as an additional tool to identify a subgroup of critically ill newborns who may respond to rescue HFOV [19,20]. A more recent study evaluating 97 newborn infants undergoing rescue HFOV in India concluded that HFOV significantly improved the oxygenation index, alveolar-arterial oxygen gradient, pH, pCO_2_, and pO_2_ and resulted in better lung recruitment within 2 hours. Fifty-seven of these infants (58.8%) survived, but the mortality rate was greater at <28 weeks gestation among babies with pulmonary hemorrhage, sepsis, and CDH [21].

The actual response to HFOV is dependent on ventilator strategy, which may vary between centers [16]. We did not observe any association between initial ventilator settings and response to rescue HFOV. This can be explained by patient care supervised by experienced neonatologists and defined open lung strategy prior to the study. The majority of neonatal studies are from old series when open lung recruitment and protective strategies were not widely used. HFOV provides the potential to maintain adequate gas exchange without imposing the large pressure swings and tidal volume changes associated with induced lung injury [15,22,23].

The only previous national multicenter study in newborn infants was published in 1999 and was performed in Spain. This Spanish multicenter study on HFOV as a salvage strategy aimed to evaluate the results of rescue HFOV in 241 newborn infants with severe lung disease in nine level III Spanish NICUs. They demonstrated that at 2 hours of HFOV, there was a significant increase in the mean PaO_2_, with concomitant decreases in FiO_2_, PaCO_2_, and OI. The in-hospital death rate was 32%. The authors reported pneumothorax (10%), interstitial emphysema (4%), IVH grades III and IV (14.5%), and CLD (35%) as side effects in their cohort and concluded that HFOV was an effective rescue strategy that improved gas exchange within 2 hours after initiation [20].

Our study had few limitations. First, it is an observational study. However, to our knowledge, the present report represents the largest multicenter study on the efficacy and safety of rescue HFOV in a variety of diseases in newborn infants. Second, although all NICUs were informed for recruitment and ventilation strategies for rescue HFOV, attendings’ follow ups might show variations because of its multicenter design and unavailability of ECMO in all centers. Third, we did not collect final cause of death data which might give more information on success of the rescue HFOV. We believe that further studies using similar large clinical databases may provide evidence regarding the efficacy of commonly used therapeutic interventions, such as rescue HFOV, in critical care that may be difficult to study with classic prospective randomized controlled trials due to expense, logistics, and clinical balance. Use of such databases will help to improve outcomes for critically ill newborn infants.

### Turkish neonatal society rescue-HFOV study group collaborators

Elif Ozyazıcı Ozkan (Division of Neonatology, Department of Pediatrics, Ondokuz Mayıs University School of Medicine, Samsun, Turkey), Sinan Uslu (Department of Neonatology, University of Health Sciences, Sisli Etfal Training and Research Hospital, Istanbul, Turkey), Nihal Demirel (Division of Neonatology, Department of Pediatrics, Yildirim Beyazıt University School of Medicine, Ankara, Turkey), Ismail Kursad Gokce (Division of Neonatology, Department of Pediatrics, Inonu University School of Medicine, Malatya, Turkey), Gonca Vardar (Department of Neonatology, University of Health Sciences, Zeynep Kamil Training and Research Hospital, Istanbul, Turkey), Münevver Türkmen (Division of Neonatology, Department of Pediatrics, Adnan Menderes University School of Medicine, Aydin, Turkey), Murat Konak, Beyza Ozan (Department of Neonatology, University of Health Sciences, Konya Training and Research Hospital, Konya, Turkey), Buket Kılıcaslan, Nejat Narlı (Neonatal Intensive Care Unit, Adana Metro Hospital, Adana, Turkey), Nihat Demir, Oguz Tuncer (Division of Neonatology, Department of Pediatrics, Yuzuncuyil University School of Medicine, Van, Turkey), Ilke Mungan Akin, Sertac Aslanoglu (Department of Neonatology, Medeniyet University Goztepe Training and Research Hospital, Istanbul, Turkey), Sebnem Calkavur, Ozgur Olukman (Department of Neonatology, University of Health Sciences, Behcet Uz Children’s Hospital, Izmir, Turkey), Bilge Tanyeri Bayraktar (Division of Neonatology, Department of Pediatrics, Bezmialem University, Istanbul, Turkey), Leyla Bilgin, Omer Guran (Department of Neonatology, University of Health Sciences, Umraniye Training and Research Hospital, Istanbul, Turkey), Meltem Aksu, Ibrahim Hirfanoglu (Division of Neonatology, Department of Pediatrics, Gazi University School of Medicine, Ankara, Turkey), Deniz Anuk Ince, Ayse N. Ecevit (Division of Neonatology, Department of Pediatrics, Baskent University School of Medicine, Ankara, Turkey), Fatma Narter (Department of Neonatology, Kartal Dr. Lutfi Kirdar Training and Research Hospital, Istanbul, Turkey)

## Supporting information

S1 File Patient data. (XLSX)
